# The Occurrence, Distribution, and Toxicity of High-Risk Ciguatera Fish Species (Grouper and Snapper) in Kiritimati Island and Marakei Island of the Republic of Kiribati

**DOI:** 10.3390/toxins14030208

**Published:** 2022-03-15

**Authors:** Jingyi Zhu, Wai-Hin Lee, Jiajun Wu, Shiwen Zhou, Ki-Chun Yip, Xiaowan Liu, Taratau Kirata, Leo-Lai Chan

**Affiliations:** 1 State Key Laboratory of Marine Pollution, Department of Biomedical Sciences, City University of Hong Kong, Kowloon Tong, Hong Kong 999077, China; jingyizhu9-c@my.cityu.edu.hk (J.Z.); waihlee-c@my.cityu.edu.hk (W.-H.L.); jiajunwu@cityu.edu.hk (J.W.); swzhou3-c@my.cityu.edu.hk (S.Z.); kicyip-c@my.cityu.edu.hk (K.-C.Y.); xiaowliu5-c@my.cityu.edu.hk (X.L.); 2 Shenzhen Key Laboratory for the Sustainable Use of Marine Biodiversity, Research Centre for the Oceans and Human Health, City University of Hong Kong Shenzhen Research Institute, Shenzhen 518057, China; 3 Ministry of Fisheries & Marine Resources Development, Kiribati Government, Tarawa 276123, Kiribati; tarataukirata@gmail.com

**Keywords:** ciguatoxin, the Republic of Kiribati, grouper, snapper

## Abstract

Ciguatera is one of the most widespread food poisonings caused by the ingestion of fish contaminated by ciguatoxins (CTXs). Snapper and grouper with high palatable and economic value are the primary food source and fish species for exportation in the Republic of Kiribati, but they are highly suspected CTX-contaminated species due to their top predatory characteristics. In this study, 60 fish specimens from 17 species of snappers and groupers collected from the Kiritimati Island and Marakei Island of the Republic of Kiribati were analyzed using mouse neuroblastoma (N2a) assay and liquid chromatography-tandem mass spectrometry (LC-MS/MS) to determine Pacific CTX-1, -2 and -3 (P-CTX-1, -2 and -3). The LC-MS/MS results show that CTXs were detected in 74.5% of specimens from Marakei Island and 61.5% of specimens from Kiritimati Island. The most toxic fish *Epinephelus coeruleopunctatus* from Marakei Island and *Cephalopholis miniata* from Kiritimati Island were detected as 53-fold and 28-fold P-CTX-1 equivalents higher than the safety level of 10 pg/g P-CTX-1 equivalents, respectively. CTX levels and composition profiles varied with species and location. The N2a results suggested that fish specimens also contain high levels of other CTX-like toxins or sodium channel activators. The distribution patterns for ciguatoxic fish of the two islands were similar, with fish sampled from the northwest being more toxic than the southwest. This study shows that groupers and snappers are high-risk species for ciguatera in the Republic of Kiribati, and these species can further be used as indicator species in ciguatera endemic areas for risk assessment.

## 1. Introduction

Ciguatera is considered to be the most widespread and non-infectious seafood-borne illness, with 10,000 to 50,000 cases per year [1]. Fish become ciguatoxic when they ingest ciguatoxins (CTXs) synthesized by bottom-dwelling dinoflagellates, such as *Gambierdiscus* and *Fukuyoa* [2]. These dinoflagellates frequently occur in shallow subtropical and tropical coastal habitats. CTXs enter the food web by herbivores grazing on macroalgae and other substrates with dinoflagellates attached to their surfaces, causing CTXs to be bio-magnified and bio-transformed along with the food web [3].

The consumption of CTX contaminated fish may cause gastrointestinal disturbance (diarrhea, nausea, vomiting, abdominal pain), cardiovascular disturbance (bradycardia, hypotension), and nervous system disorders (perioral and systemic sensory abnormalities) after a toxic meal [4]. More seriously, repeated exposure to CTX may aggravate symptoms, and death may occur after eating the internal organs (e.g., viscera) of ciguatoxic fish [5]. CTX cannot be removed or rendered harmless by cooking or freezing due to their colorless, odorless, tasteless, and heat-stable nature [2]. Therefore, current protection against ciguatera fish poisoning (CFP) includes avoiding the sale of high-risk fish species that are known to accumulate CTXs or the fish species from known toxic sites. Currently, more than 400 fish species are identified as ciguatoxic (e.g., moray eels, groupers, and snappers), and different accumulation capacities for CTXs were found among different species [6,7]. These toxic fish are usually bottom dwellers, and their toxicity depends mainly on their location [8].

More frequent incidents of ciguatera poisoning occurred in the central Pacific than in any other region on earth [9]. One of its nations, the Republic of Kiribati, has an incidence rate almost five times higher than the rest of the Pacific region [10,11,12]. The republic of Kiribati comprises 32 atolls and is divided into three island groups: the Gilbert Islands, the Phoenix Islands, and the Line Islands, with a population of more than 110,000 [13]. Due to the limited land area and natural resources, Kiribati islanders rely heavily on fishery resources for food (contributing an average of 55.8% of dietary protein) and economic income from seafood exports and fishing [14]. Therefore, the loss of fishing areas resulting from ciguatera has a significant impact on the well-being of Kiribati Islanders. Marakei Island is the second-largest island in the northern Gilbert Islands and is one of the most populated islands with high ciguatera incidence [14]. Many studies demonstrated that most of the fish specimens from the Marakei atoll are ciguatoxic, especially in the western region of the island [3,15]. The Kiritimati (Christmas) Atoll is a partially filled-bucket atoll in the Northern Line Islands and accounts for over half of the total land area of the nation [16]. The environment of Kiritimati is more diverse compared with the other atolls of Kiribati owing to the large land area, relatively small central lagoon, and a large number of smaller lagoons [15]. Ciguatera first appeared on Kiritimati between 1958 and 1962 and has remained present as evidenced by medical records. However, to the best of our knowledge, no relevant research was conducted on CFP in Kiritimati.

Groupers and snappers are essential fishery resources with high economic and nutritional values in the major international markets. They are one of the most popular export fish species in the market, supporting the livelihoods and food security of fishing communities worldwide because of their excellent meat quality and high demands [17]. However, they are mainly piscivorous and are potentially contaminated by CTXs due to their high tropic level. These two fish species can accumulate large quantities of CTXs because of their slow growth, long life span, epibenthic behavior, and high location fidelity features [18,19]. Many studies demonstrated that groupers and snappers were generally more toxic than other fish species, where the CTX levels of *Cephalopholis argus* and *Epinephelus spilotoceps* were reported to exceed the safety level (10 pg P-CTX-1 equiv/g) by 1000 times [3,6]. To date, a comprehensive study on the occurrence, distribution, and toxicity of CTXs in different species of snappers and groupers in Kiribati is still limited.

Liquid chromatography-tandem mass spectrometry (LC-MS/MS) analysis and the mouse neuroblastoma (N2a) assay are widely applied in determining the levels of CTXs in contaminated fish samples. The European Food Safety Authority (EFSA) recommend in vitro assays for toxin screening and LC-MS/MS analysis for toxin confirmation [20]. Although LC-MS/MS analysis is high in sensitivity and specificity compared to the N2a assay, it can only detect the known CTX toxins with commercially available toxin standards. However, the N2a assay can detect all CTX analogues and other sodium channel-enhancing/blocking toxins. Therefore, in this study, N2a and LC-MS/MS were used to determine the Pacific CTX-1, -2 and -3 (P-CTX-1, -2 and -3) in groupers and snappers from Kiritimati Island and the Marakei Islands. This study could provide quantitative information for the safety management of these fisheries and ensure that these resources can continue to provide food and revenue for the Republic of Kiribati.

## 2. Results

### 2.1. Concentrations and Composition Profiles of CTXs

#### 2.1.1. CTX concentrations and Composition Profiles in Marakei Island

**Figure 1:**
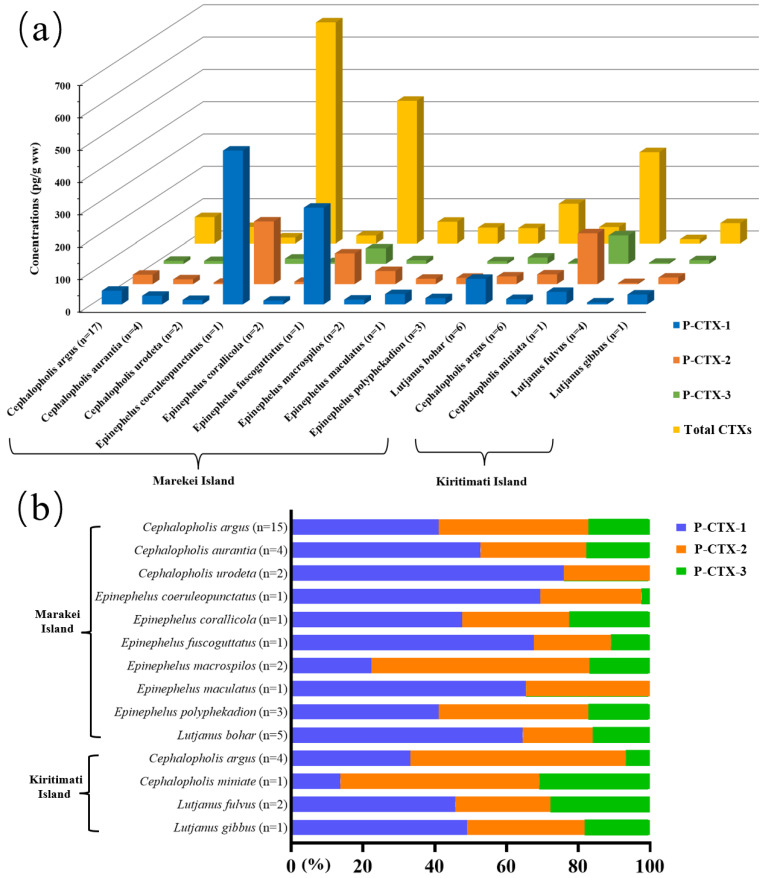
(**a**) Mean concentrations of P-CTX-1, -2, -3 and total CTXs, and (**b**) composition profiles of groupers and snappers with detectable CTX levels from Marakei Island and Kiritimati Island.

CTXs were detected in 10 fish species from Marakei Island, and total CTX levels in fish samples ranged from <LOQ (limit of quantification) to 683 pg/g (Figure 1a). *E. coeruleopunctatus* (*n* = 1), *E. fuscoguttatus* (*n* = 1), and *L. bohar* (*n* = 6) were the three most toxic fish species with total mean CTX concentrations of 683, 441, and 122 pg/g, respectively. P-CTX-1, -2, and -3 levels in those fish were in the range of <LOQ − 474, <LOQ − 192, and <LOQ − 75.0 pg/g, respectively (Table S1). The grouper *E*. *coeruleopunctatus* had the highest levels of P-CTX-1 (474 pg/g) and P-CTX-2 (192 pg/g). The occurrence of P-CTX-1 constituted 74.5% of all fish species from Marakei, which was higher than P-CTX-2 (63.8%) and P-CTX-3 (42.6%) (Table S2). Eight out of ten fish species were detected for all CTXs (P-CTX-1, 2, and -3), and the remaining two species (*E. maculatus* and *C. urodeta*) showed only P-CTX-1 and 2. P-CTX-1, 2, and 3 accounted for 22.2–76.0% (median 58.6%), 19.4–60.8% (median 29.6%), and 0–17.8% (median 16.5%) of total CTXs, respectively (Figure 1b). P-CTX-1 was the dominant P-CTXs in *C. aurantia*, *C. urodeta*, *E. coeruleopunctatus*, *E. corallicola*, *E. fuscoguttatus*, *E. maculatus*, and *L. bohar*, contributing to 52.8%, 76.0%, 69.4%, 47.6%, 67.6%, 65.5%, and 64.6% of the total CTXs, respectively. The fish species, *C. argus*, *E. macrospilos,* and *E. polyphekadion*, were dominated by P-CTX-2, accounting for 41.7%, 60.8%, and 41.7%, respectively. However, no CTXs were detected in five species, including *E. areolatus*, *E. hexagonatus*, *E. merra*, *E. tauvina,* and *L. fulvus*.

#### 2.1.2. CTX Concentrations and Composition Profiles in Kiritimati Island

For Kiritimati Island, CTXs were detected in 8 out of 13 fish specimens with the total P-CTXs levels in the range of 5.90 to 282 pg/g, and their P-CTX-1, -2, and -3 levels were 4.10–76.7, <LOQ − 156, and <LOQ − 87.1 pg/g, respectively (Figure 1a). The maximum concentrations of P-CTX-1, -2, and -3 among all fish species from Kiritimati Island were 76.7 pg/g of *C. argus* and 156 and 87.1 pg/g of *C. miniate*, respectively (Table S1). The occurrence of total CTXs accounted for 61.5% of all fish specimens from Kiritimati, which was slightly lower than that of Marakei (70.2%), while the P-CTX-1, -2, and -3 occurrence rates were 61.5%, 53.9%, and 46.2%, respectively. The composition profiles from Kiritimati Island were less diversified than Marakei Island. The profiles of all fish species from Kiritimati were composed of P-CTX-1, 2, and 3 (Figure 1b). The proportions of P-CTX-1, 2, and 3 were 13.7–49.0% (median 39.5%), 26.4–60.1% (median 44.0%), and 6.7–30.9% (median 23.0%), respectively. The CTX profiles of the *C. argus* and *C. miniata* were dominated by P-CTX-2 with proportions of 60.1% and 55.4%, respectively, while *L. fulvus* and *L. gibbus* were dominated by P-CTX-1 with proportions of 45.8% and 49.0%, respectively.

#### 2.1.3. Relationship between Body Size (Total Length and Body Weight) and CTX Levels of Grouper and Snapper from Marakei Island and Kiritimati Island

The relationship between CTX levels and total length and body weight of toxic fish was analyzed by linear regression. The information of total length and body weight of fish samples are shown in Table S1. This analysis was attempted to correlate CTX levels with different fish sizes for several fish species obtained from Marakei Island and Kiritimati Island. However, no correlation was found between the CTX concentrations and total length or bodyweight among species for both islands.

### 2.2. Ciguatoxicity Determined by N2a and LC-MS/MS from Marakei Island and Kiritimati Island

The ciguatoxicities of grouper and snapper determined by N2a and LC-MS/MS from Marakei Island and Kiritimati Island can be shown in Table 1. The ciguatoxicity (P-CTX-1 eq.) was calculated by the sum of the concentrations of P-CTX-1, 2, and 3 determined by LC-MS/MS multiplied by their respective toxicity equivalent factors (TEFs) (1 for P-CTX-1, 0.3 for P-CTX-2, and 0.3 for P-CTX-3, respectively) [3]. Ciguatoxicities determined by LC-MS/MS in fish species contributed to 0–76.1% (median 39.1%) for Marakei Island and 0–48.2% (median 34.2%) for Kiritimati Island of the total ciguatoxicities obtained by N2a, respectively. Total ciguatoxicity for all fish specimens determined by LC-MS/MS had a good correlation with the data from the N2a (R^2^ = 0.9566, y = 0.3308x − 2.4733). Higher frequency of CTXs could be found in fish specimens determined by N2a (95.7% for Marakei Island and 92.3% for Kiritimati Island) compared to LC-MS/MS (74.5% for Marakei Island and 61.5% for Kiritimati Island).

### 2.3. Spatial Distribution of Ciguatoxic Grouper and Snapper from Marakei Island and Kiritimati Island

#### 2.3.1. Ciguatoxicities of Fish Samples Determined by N2a and LC-MS/MS in Marakei Island

**Figure 2:**
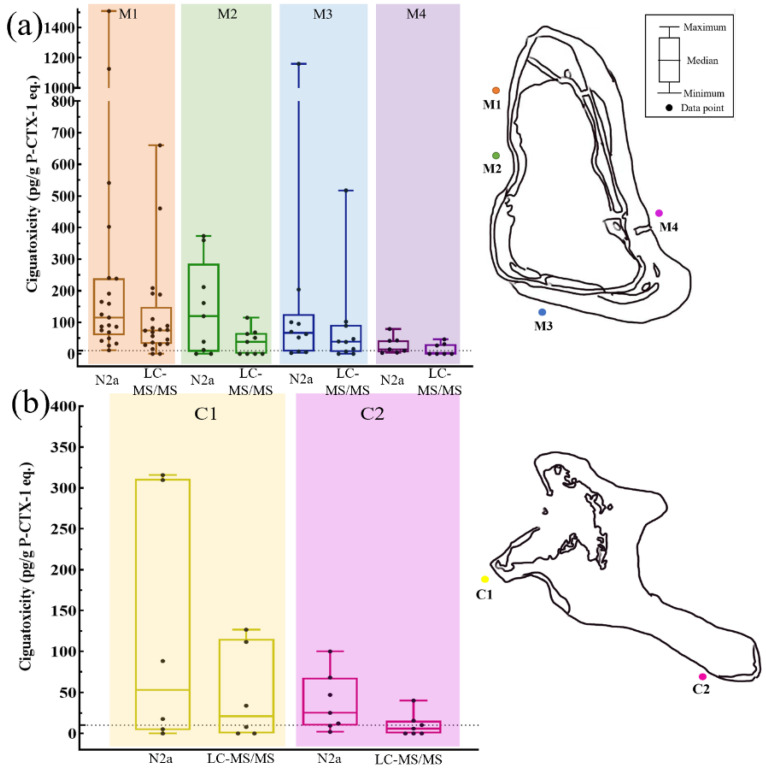
(**a**) Ciguatoxicities of fish samples determined by LC-MS/MS and N2a in Marakei Island. (**b**) Ciguatoxicities of fish samples determined by LC-MS/MS and N2a in Kiritimati Island. (The dotted line represents the safety level of 10 pg/g P-CTX-1 equivalents.).

The ciguatoxicities of fish samples in Marakei Island determined by LC-MS/MS and N2a can be shown in Figure 2a. Fish samples collected from sampling site M1 were significantly more toxic than those from M4 (*p* < 0.05, Mann—Whitney U test). The average ciguatoxicity of fish samples determined by LC-MS/MS in Marakei Island ranked from high to low was M1(86.9 pg/g P-CTX-1 eq.), M3(62.3 pg/g P-CTX-1 eq.), M2 (24.3 pg/g P-CTX-1 eq.), and M4 (9.00 pg/g P-CTX-1 eq.), respectively. The results for N2a were in the order of site M1 (256 pg/g P-CTX-1 eq.), site M3 (175 pg/g P-CTX-1 eq.), site M2 (141 pg/g P-CTX-1 eq.), and site M4 (27.6 pg/g P-CTX-1 eq.). Based on the safety threshold of 10 pg/g P-CTX-1 equivalents, 90.5%, 55.6%, 70%, and 42.9% of fish specimens determined by LC-MS/MS were ciguatoxic within areas M1, M2, M3, and M4, respectively, while 100%, 77.8%, 70% and 57.1% of fish at site M1, M2, M3 and M4 by N2a were found to be unsafe, respectively.

#### 2.3.2. Ciguatoxicities of Fish Samples Determined by N2a and LC-MS/MS in Kiritimati Islands

Based on the results (Figure 2b), there was no significant difference in the toxicities of fish samples between the two sampling sites (C1 and C2) (*p* > 0.05). Among the specimens, the average ciguatoxicity of the fish from site C1 (122 pg/g P-CTX-1 eq. for N2a and 46.7 pg/g P-CTX-1 eq. for LC-MS/MS) was higher than site C2 (37.7 pg/g P-CTX-1 eq. for N2a and 10.3 pg/g P-CTX-1 eq. for LC-MS/MS). In total, 66.7% and 71.4% of fish at sites C1 and C2 of Kiritimati Island determined by N2a were found to have P-CTX-1 equivalent toxicity above the safety threshold, respectively. For LC-MS/MS results, 50% and 42.9% of fish samples at sites C1 and C2 were considered unsafe for human consumption, respectively.

## 3. Discussion

Kiribati Islands have limited land-based resources, so islanders heavily rely on marine resources. Fish are an essential nutritional and gastronomic source for the people in the Republic of Kiribati and play a vital role in subsistence and commerce. Grouper and snapper are high in nutritional and commercial value but are prohibited or restricted for sale and consumption in some areas due to their CTX accumulation capacity [8]. This study investigated the occurrence, distribution, and toxicity of high-risk ciguatera fish species (17 species groupers and snappers) from Marakei Island and Kiritimati Island of the Republic of Kiribati. 

### 3.1. CTX Levels in Ciguateric Groupers and Snappers of Marakei Island and Kiritimati Island

In this study, only CTXs (P-CTX-1, -2 and -3) were analyzed in carnivorous fish samples. P-CTX-1 (CTX1B) was found to be high in concentration and the major in CTXs in carnivorous fish (such as grouper and snapper) in the Republic of Kiribati [3]. Other CTX congeners (e.g., CTX-4A and -B) are more likely to accumulate in herbivores [21]. Moreover, in a previous study, toxin profiles of fish from Okinawa showed that CTX1B was the major toxin among CTX1B and CTX3C congeners [22]. P-CTX-2 and -3 can be oxidized into the more toxic and polar P-CTX-1 in carnivorous fish [23]. Therefore, in this study, the levels of P-CTX-1, -2, and -3 were determined in fish samples from the Republic of Kiribati.

Total CTX levels (P-CTX-1, -2, and -3) in fish samples from Marakei Island ranged from <LOQ to 683 pg/g with high occurrence (74.5%), and their P-CTX-1, -2, and -3 levels were in the range of <LOQ − 474, <LOQ − 192, and <LOQ − 75.0 pg/g, respectively (Figure 1a). *E. coeruleopunctatus*, *E. fuscoguttatus,* and *L. bohar* were the most toxic fish species. These three fish species generally belong to large fish species, which are the most fished species for human consumption worldwide and were previously found to be unsafe in Japan, French Polynesia, and the republic of Kiribati [24,25,26,27]. The occurrence of total CTXs in *C. argus* (*n* = 17) accounted for 88.2%. This result was consistent with previous studies that *C. argus* was potentially CTXs contaminated fish species [3,6,28]. 

This is the first report of CTX levels for the fish from Kiritimati Island, and 61.5% of fish samples could be detected to contain CTXs (Table S1). The levels of total CTXs were in the range of <LOQ to 282 pg/ g, and their P-CTX-1, -2, and -3 levels were <LOQ − 76.7, <LOQ − 156, and <LOQ − 87.1 pg/g, respectively. The most toxic fish species were *C. miniata* (total CTXs 282 pg/g, *n* = 1). A previous study showed that *C. miniata* were usually involved in intoxication events of CFP in the Pacific [29]. 

Grouper and snapper are characterized by their long lifespan and slow growth rate. A previous study found that slow-growing fish may have higher CTX concentrations than fast-growing ones [29]. Moreover, food choice plays a vital role in determining ciguatoxicity in fish. Previous studies suggested that diet may influence CTX levels and composition profiles in food web components [23]. The details of food items of fish species are shown in Table S3. The diets of groupers and snappers are composed of various prey items, mainly fish and macroinvertebrates, to different extents. It was reported that piscivorous fish species of higher trophic levels are more likely to accumulate CTXs [30]. Overall, these features can explain why most groupers and snappers accumulated higher concentrations of CTXs.

### 3.2. Ciguatoxicity of Grouper and Snapper Determined by N2a and LC-MS/MS 

Based on the LC-MS/MS results, 72.3% of fish specimens from Marakei Island and 46.2% from Kiritimati Island can lead to CFP (above 10 pg/g P-CTX-1 equivalents), respectively (Figure 2). This is consistent with previous findings that Kiribati Islands have a high incidence of CFP [3,6,14]. The most toxic individual grouper *E. coeruleopunctatus* and snapper *L. bohar* from Marakei Island were found to contain P-CTX-1 equivalents 53-fold and 9-fold higher than the safety level, respectively. For Kiritimati Island, the most toxic individual *C. miniata* was found to exceed the toxicity threshold by 28-fold. The high frequency and CTX levels of ciguateric groupers and snappers were found in Kiribati show that they were unsafe for human consumption and pose a threat to seafood safety and the development of the fishing industry in Kiribati.

Total ciguatoxicity determined by LC-MS/MS has a good correlation with the data from the N2a. The occurrence of ciguatoxic fish accounted for 95.7% and 92.3% of the total fish samples from Marakei Island and Kiritimati Island, respectively (Table 1). In total, 83% (Marakei Island) and 69.2% (Kiritimati Island) of fish specimens exceeded the safety threshold, respectively. The ciguatoxicities determined by N2a were approximately 3.2 times higher than the levels determined by LC-MS/MS. N2a can also detect other CTX congeners and sodium channel activators, such as brevetoxins, that share the same receptor site on the neuronal channel protein, affecting the measured value [31]. Therefore, N2a results suggested that grouper and snapper are not only high-risk CFP species in Kiribati but also contain high levels of other CTX-like toxins or sodium channel activators. Further study is needed to determine the levels of other CTX congeners and sodium channel activators.

### 3.3. Relationship between Body Size and CTX Levels of Groupers and Snappers from Marakei Island and Kiritimati Island

In this study, there is no obvious correlation between CTX concentrations and total length or weight among 17 fish species in Kiribati. These results were consistent with previous research from Fakarava Island and Hawaii [10,25]. Mak et al. found that the heaviest groupers contained the greatest total CTX concentrations, showing a positive relationship between toxicity and body size [3]. This result was inconsistent with this study, where the total CTX levels of the heaviest two-spotted red snapper (4400 g, 50.5 pg/g) were 13.5-fold lower than that in the white-spotted grouper (608 g, 683 pg/g). For the blue-spotted groupers collected from Marakei Island (*n* = 17), there was no significant relationship between body size and CTX levels. The possible reason is that the accumulation of CTXs may be related to location-fidelity, age, the capability of toxin storage and clearance, and growth rate, not just body size [32,33]. 

The effects of CTXs on fish may vary among different fish species. It was reported that CTXs influence fish recruitment because CTXs may cause behavioral and morphological changes in sensitive fish species [3]. Thus, they are easy prey for predators and are killed directly by CTXs. This can explain why the limited amount of CTXs accumulated in certain fish species and certain levels of CTXs may be lethal to fish. Therefore, it is difficult for toxic fish to grow to their maximum length. The information of maximum length for fish species is shown in Table S3. In this study, no toxic fish individual with maximum length could be found. However, high levels of CTXs can be accumulated in certain fish species capable of posing a threat to human health.

### 3.4. Composition Profiles of Ciguatoxic Groupers and Snappers from Marakei Island and Kiritimati Island

P-CTX-1 was the dominant CTXs in most species in Marakei Island, compared to other CTX1B analogues (Figure 1b). This result was consistent with previous studies suggesting that P-CTX-1 was the primary CTXs in carnivorous fishes [34,35]. The composition profiles from Kiritimati Island were less diversified than Marakei Island due to limited sample sizes. Groupers and snappers are top predators, and they would contain the most oxidized and toxic CTX derivatives [23]. A previous study demonstrated the higher capacity of groupers and snappers to oxidize P-CTX-2,3 and 4A/4B into the more potent P-CTX-1 [21]. The greater concentration of potent P-CTX-1 present in most fishes further confirmed that groupers and snappers are high-risk CFP species in Kiribati. 

Toxin profiles were found to vary between different fish species. A possible explanation is that toxins in fish species transformed in a species-specific manner, and some fish species are more likely to accumulate certain toxin analogues [36]. Another possible explanation is that composition profiles were also related to the eating habits of the fish species. Piscivorous fish can increase the chances of accumulating P-CTX-1, while fish that mainly feed on cephalopods and crustaceans can increase the probability of accumulating CTX-2/3 [3,29]. Different toxicity patterns were observed among individuals of the same fish species. For example, the dominant CTXs for *C. argus* (*n* = 19) collected from six sampling points differed. A previous study suggested that toxin profiles were site-specific and may be related to toxin congeners in microalgae species existing in this area, resulting in different composition profiles in the same species [23,36]. 

### 3.5. Spatial Distribution of Ciguatoxic Groupers and Snappers from Marakei Island and Kiritimati Island

Ciguatoxicities of fish samples in groupers and snappers at six sampling points of Marakei Island and Kiritimati Island are shown in Figure 2. For Marakei Island, the average ciguatoxicities of fishes were in the order of site M1 > site M3 > site M2 > site M4. Site M1, located at the northernmost point of the island, was the most toxic site with a high frequency of toxic fish, and the average toxicity was 1.5-fold and 10-fold higher than the southern site M3 and eastern site M4, respectively. This result corresponds well to previous studies that the first ciguatoxic fishes were caught from the Rawanawi village (near site M1) and expanded further southward, and the abundance of toxic dinoflagellate was higher in the north [14,33]. This distribution pattern of ciguatoxic fish supported that CFP intoxication events frequently occurred in the northwestern side of the island [6,37]. More surprisingly, the ciguatoxicity of fish in the four sampling sites varied as much as 220 times within species, suggesting that locations play a vital role in determining whether the fish have CTXs. For Kiritimati Island, fish samples from the southern site C2 were less toxic than the northern site C1, and the average toxicity was 4.2-fold lower than site C1. It was observed that fish toxicity was higher in the northwest than in the southwest of the two islands. The trade wind direction is northeast, east, and southeast, which may cause high water turbulence in this area [38,39]. Trade winds influenced site M1 of Marakei Island and site C1 of Kiritimati Island less than other sampling sites. Thus, relative calm water can contribute to the colonization and proliferation of dinoflagellates and the accumulation of ciguatoxicity [14]. The differences in fish toxicity of six sampling points may also be linked to regional variations in CFP-associated dinoflagellates either in terms of species composition or strain toxicity in ciguatoxin endemic areas [40].

## 4. Conclusions

Overall, a high frequency of ciguatoxic snappers and groupers were found in the Marakei and Kiritimati Islands, and most of them were not safe for human consumption. CTX levels and composition profiles varied with species and locations. No correlation between CTX concentrations and the total length or weight of fish specimens was found. Fish from the northwestern sampling site of Marakei Island were more toxic than other sites, and a similar distribution pattern can be observed for Kiritimati Island. Additional studies are suggested to investigate the effect of age, feeding habits, and metabolic pathways on the accumulation of ciguatoxins in ciguatoxic fishes. 

## 5. Materials and Methods

### 5.1. Sample Collection

**Figure 3:**
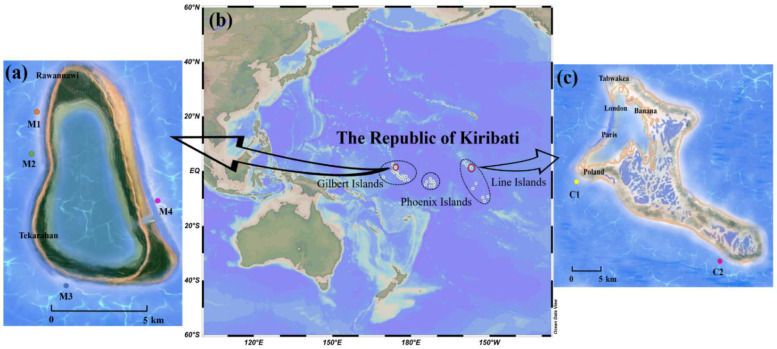
(**a**) Location of the sampling sites of Marakei Islands; (**b**) location of the Republic of Kiribati; (**c**) location of the sampling sites of Kiritimati Islands.

The Republic of Kiribati, situated in the Central Pacific basin, comprises three island groups: the Gilbert Islands, the Phoenix Islands, and the Line Islands (Figure 3b). The Marakei atoll (Figure 3a) is the second-largest island in the northern Gilbert Islands, and the Kiritimati (Christmas) atoll (Figure 3c) is in the Northern Line Islands. A total of 60 fish samples, which could be categorized into 17 species, were collected from the Kiritimati and Marakei atolls in the years 2019 and 2014, respectively. The detailed information of the groupers (Serranidae) and snappers (Lutjanidae) is provided in Table 2. All the fish samples were kept at −20 °C until analysis. Fish samples were identified, measured, and weighed in the laboratory, and then the toxicities of individual fish were tested to compare the CTX levels. 

### 5.2. CTXs Extraction Method 

Target CTXs in fish samples were extracted according to previous methods, and the fish samples were studied in duplicate [41]. P-CTX-1 (CTX1B), P-CTX-2 (52-epi-54-deoxy CTX1B), and P-CTX-3 (54-deoxy CTX1B) (purity > 95%) were purchased from Professor R. Lewis (The University of Queensland, Brisbane, Australia). The chemical structures of P-CTX-1, -2, and -3 are shown in Figure S1. In total, 5 g dorsal muscle samples were freeze-dried overnight by a vacuum dryer (Labconco FreeZone, Kansas, MO, USA) to remove moisture. Each sample was then mixed with 3.5 g diatomaceous earth (as a dispersant and drying agent) and then transferred into a 22 mL stainless steel extraction cell with two Whatman glass fiber filters at the bottom for further extraction. CTXs were extracted using an ASE 200 system (Dionex, Sunnyvale, CA, USA) at 75 °C and 1500 psi for 5 min of heating, followed by two static extractions with methanol. The final extracts were concentrated to almost dryness under a gentle stream of high-purify N_2_ and further dissolved in 15 mL 50% methanol/MilliQ water before the clean-up procedure.

### 5.3. CTXs Clean-Up Procedure

Solid-phase extraction (SPE) was used for further clean-up. The InertSep C18 cartridges (GL science, 500 mg, 6 mL) were preconditioned successively with 15 mL of methanol and 10 mL of Milli-Q water followed by washing with 6.5 mL of 65% methanol/MilliQ water. Afterward, the target analyte was eluted with 12 mL of 80% methanol/MilliQ water. In total, 10 mL of CHCl_3_ and 6.3 mL of 1 M NaCl were mixed well with the target analyte. The lower chloroform layer was collected and dried by high-purify N_2_. The residue was dissolved in 4 mL of CHCl_3_ before loading on a Sep-Pak silica cartridge (Waters, 500 mg, 6 mL). The silica cartridges were preconditioned with 30 mL CHCl_3_ and washed with 4.5 mL of CHCl_3_. The target analyte was eluted with 8 mL of 90% CHCl_3_ in methanol. The residue was resuspended with 100 µL methanol for instrumental analysis after being dried by high-purify N_2_.

### 5.4. Determination of CTX Concentrations

#### 5.4.1. Mouse Neuroblastoma (N2a) Assay 

An N2a cell cytotoxicity assay was conducted using previously reported methods [6,42]. N2a cells (CCL131; ATCC, Manassas, VA, USA) were cultured in RPMI-1640 medium (Gibco, Life Technologies, Carlsbad, CA) supplemented with 10% fetal bovine serum (BD Biosciences, San Jose, CA, USA) at 37 °C in 5% CO_2_. In total, 2 g/L Na_2_CO_3_ and 50 units/mL penicillin and 50 µg/mL streptomycin and 2.5 µg/mL Fungizone^®^ (Gibco Life Technologies, Carlsbad, CA, USA) were added to the culture. N2a cells were seeded into 96-well culture plates (excluding peripheral wells) at a cell density of 2.5 × 10^5^ cells/mL with 200 μL of the medium. The peripheral wells were filled with 300 μL PBS to avoid edge effects. After incubating for 24 h, the medium was renewed with complete RPMI-1640 (including 0.1 mM ouabain and 0.01 mM veratridine). A total of 10 µL/well P-CTX-1 standards were added to the cells at eight concentrations ranging from 2.438 pg/mL to 312 pg/mL in six replicates. Sample extracts (in 20% methanol in PBS) were diluted and tested in triplicate. The final volume of all wells was 200 μL. After incubating for 18 h, the assay evaluated cell viability by MTT (3-(4,5-dimethylthiazol-2-yl)-2,5-diphenyltetrazolium). Before measuring absorbance, 50 μL isopropanol with 0.1 M HCl was added. Absorbance was measured at 595 nm using a microplate reader with a reference wavelength of 655 nm. The optical density obtained from each well was normalized by MTT blank. Ciguatoxicity values of the fish samples were determined from the standard curve. The LOQ of the method was 4.875 pg/g. The assays were performed twice, and the ciguatoxicity values are reported as mean P-CTX-1 equivalents between the two assays. The inter-plate relative standard deviation was 23.2%, and the inter-assay relative standard deviation was 28.5%. The P-CTX-1 standard curve for N2a can be shown in Figure S2.

#### 5.4.2. Liquid Chromatography-Tandem Mass Spectrometry (LC-MS/MS)

The determination of CTXs was performed using LC-MS/MS consisting of an Agilent 1290 UPLC system (Agilent, Palo Alto, CA, USA) and interfaced with a 5500 QTRAP system (AB Sciex, Foster City, CA, USA) with a turbo-ion spray operating in the positive ion and multiple-reaction-monitoring mode. The injection volume was 10 μL. An InertSustain QA-C18 column (100 × 2.1 mm i.d., 1.9 μm) was used for the separation. Gradient elution was performed at a flow rate of 200 μL/min with (A) Milli-Q water containing 0.1% formic acid and 2 mM ammonium formate and (B) 95% acetonitrile in Milli-Q water, containing 0.1% formic acid and 2 mM ammonium formate. The initial gradient condition started at 80% mobile phase B and was maintained for 9 min. It was then ramped to 100% mobile phase B in 1 min. 100% mobile phase B was held for 4 min before returning to 80% organic mobile phase in 0.1 min. The column was then equilibrated for 4.9 min with the 80% mobile phase B before the next sample injection. Further details of CTXs and mass spectrometer operating parameters are shown in Table S4.

#### 5.4.3. Quality Assurance and Control

Matrix spike recoveries were performed by spiking 0.1 ng CTXs (P-CTX-1, -2, and -3) into the freeze-dried muscle of non-toxic mangrove grouper muscle (*n* = 3). Matrix spike recoveries were 80.4–89.3% for P-CTX-1, 78.8–80.4% for P-CTX-2, and 70.6–83.8% for P-CTX-3, with RSDs less than 10%. The calibration curves of CTXs were generated from the standard solution with seven concentrations ranging from 0.156 to 10 ppb for P-CTX-1, eight concentrations from 0.078 to 10 ppb for P-CTX-2 and 3. “1/x” weighted linear regression analysis with a correlation coefficient better than 0.990 was used. The quantification of CTXs was based on the calibration curve using the [M + H − 2H_2_O] + peak area, compared with the corresponding CTX concentrations. The limits of quantification (LOQs) were determined by analyzing the lowest CTX concentrations at a signal-to-noise ratio of 10:1. The LOQ for P-CTX-1, -2, and -3 were 3.12, 3.12, and 1.56 pg/g, respectively. The representative chromatograms of a contaminated sample after dilution can be shown in Figure S3.

### 5.5. Data Analysis

Statistical analysis was conducted using Kolmogorov–Smirnov tests for evaluating the normality of data (IBM SPSS Statistics version 23.0, SPSS Inc., Chicago, IL, USA). The Student’s *t*-test was used if data passed normality. Otherwise, nonparametric Mann–Whitney U tests were used. Linear regression was used to analyze the relationship between body size and CTX levels and the relationship between the CTXs levels obtained by N2a and LC-MS/MS. Statistical significance was set at *p* < 0.05. 

## Figures and Tables

**Table 1:** Ciguatoxicity (pg/g P-CTX-1 eq.) of grouper and snapper determined by LC-MS/MS and N2a.

Fish Species	Number of Individuals (*n*)	Sample Site	N2a Results		LC-MS/MS Results	
			Occurrence	Mean Toxicity ± SD (pg/g P-CTX-1 eq.)	Occurrence	Mean Toxicity ± SD (pg/g P-CTX-1 eq.)
Marakei Island						
*Cephalopholis argus*	17	M1, M2, M4	100%	158 ± 137	88.2%	54.2 ± 42.0
*Cephalopholis aurantia*	4	M3, M4	100%	87.4 ± 78.1	100%	33.9 ± 31.5
*Cephalopholis urodeta*	2	M1, M3	100%	19.7 ± 17.6	100%	15.0 ± 11.7
*Epinephelus areolatus*	1	M4	50%	3.00	0%	ND
*Epinephelus coeruleopunctatus*	1	M1	100%	1508	100%	537
*Epinephelus corallicola*	2	M4	75%	41.3 ± 52.8	50%	15.4 ± 21.7
*Epinephelus fuscoguttatus*	1	M1	100%	1126	100%	341
*Epinephelus hexagonatus*	1	M2	0%	ND	0%	ND
*Epinephelus macrospilos*	2	M3	100%	76.1 ± 34.3	100%	30.4 ± 12.7
*Epinephelus maculatus*	1	M3	100%	95.1	100%	36.9
*Epinephelus merra*	2	M1, M3	75%	14.4 ± 16.8	0%	ND
*Epinehelus polyphekadion*	3	M1, M2	100%	92.3 ± 62.0	100%	27.6 ± 16.3
*Epinephelus tauvina*	2	M2	50%	19.1 ± 27.0	0%	ND
*Lutjanus bohar*	6	M2, M3	91.7%	364 ± 416	75%	92.1 ± 155
*Lutjanus fulvus*	2	M1, M3	100%	8.30 ± 4.20	0%	ND
Total			95.7%		74.5%	
Kiritimati Island						
*Cephalopholis argus*	6	C1, C2	75%	74.1 ± 118	66.7%	26.7 ± 49.3
*Cephalopholis miniata*	1	C1	100%	315	100%	111
*Lutjanus bohar*	1	C2	100%	9.50	0%	ND
*Lutjanus fulvus*	4	C1, C2	100%	32.7 ± 37.9	50%	9.90 ± 16.2
*Lutjanus gibbus*	1	C2	100%	100	100%	40.2
Total			92.3%		61.5%	

ND: not detected.

**Table 2:** List of groupers and snappers collected from Marakei Island and Kiritimati Island in the Republic of Kiribati. Common name and trophic level are from FishBase (https://www.fishbase.se/search.php, accessed on 1 February 2022).

Species	Abbreviation	Common Name	Number of Individuals (*n*)	Sampling Site	Trophic Level
*Cephalopholis argus*	*C. argus*	Blue-spotted grouper	23	M1, M2, M4, C1, C2	4.5
*Cephalopholis aurantia*	*C. aurantia*	Golden hind	4	M4, M3	4
*Cephalopholis miniata*	*C. miniata*	Coral hind	1	C1	4.3
*Cephalopholis urodeta*	*C. urodeta*	Darkfin hind	2	M1, M3	4
*Epinephelus areolatus*	*E. areolatus*	Areolate grouper	1	M4	3.7
*Epinephelus coeruleopunctatus*	*E. coeruleopunctatus*	White-spotted grouper	1	M1	3.7
*Epinephelus corallicola*	*E. corallicola*	Coral grouper	2	M4	3.8
*Epinephelus fuscoguttatus*	*E. fuscoguttatus*	Brown-marbled grouper	1	M1	4.1
*Epinephelus hexagonatus*	*E. hexagonatus*	Starspotted grouper	1	M2	4.1
*Epinephelus macrospilos*	*E. macrospilos*	Snubnose grouper	2	M3	3.8
*Epinephelus maculatus*	*E. maculatus*	Highfin grouper	1	M3	4
*Epinephelus merra*	*E. merra*	Honeycomb grouper	2	M1, M3	3.8
*Epinephelus polyphekadion*	*E. polyphekadion*	Camouflage grouper	3	M1, M2	4
*Epinephelus tauvina*	*E. tauvina*	Greasy grouper	2	M2	4.1
*Lutjanus bohar*	*L. bohar*	Two-spot red snapper	7	M2, M3, C2	4.3
*Lutjanus fulvus*	*L. fulvus*	Blacktail snapper	6	M1, M3, C1, C2	3.6
*Lutjanus gibbus*	*L. gibbus*	Humpback red snapper	1	C1	4.1

## Data Availability

Data are available upon request, please contact the first author Jingyi Zhu.

## Supplementary Materials

The following supporting information can be downloaded at: https://www.mdpi.com/article/10.3390/toxins14030208/s1, Figure S1: Chemical structures of (a) P-CTX-1, (b) P-CTX-2, and (c) P-CTX-3; Figure S2: P-CTX-1 standard curve for N2a; Figure S3: Representative chromatograms of contaminated sample after dilution (*Epinephelus coeruleopunctatus*); Table S1: Total length (cm), body weight (g) and concentrations of CTXs (pg/g ww); Table S2: Concentrations of CTXs of grouper and snapper from Marakei Island and Kiritimati Island determined by LC-MS/MS; Table S3: List of maximum length, trophic level, and food items of fish from FishBase; Table S4; LC-MS/MS parameters and retention times for P-CTX-1, -2 and -3.
