# Supplementary material for: The Occurrence, Distribution, and Toxicity of High-Risk Ciguatera Fish Species (Grouper and Snapper) in Kiritimati Island and Marakei Island of the Republic of Kiribati
Source: Toxins (Basel). 2022 Mar 15;14(3):208. doi: 10.3390/toxins14030208 (PMC8952361; doi:10.3390/toxins14030208)
Supplement: Supplementary file 1 [file toxins-14-00208-s001.zip › toxins-1598730-supplementary.pdf]

The Occurrence, Distribution, and Toxicity of High-risk Ciguatera fish species (Grouper and Snapper) in Kiritimati Island and Marakei Island of the Republic of Kiribati

Table S1 Total length (cm), body weight (g) and concentrations of CTXs (pg/g ww).

Species	Label	Location	Total length (cm)	Body weight (g)	P-CTX-1 Mean ± SD (pg/g)	P-CTX-2 Mean ± SD (pg/g)	P-CTX-3 Mean ± SD (pg/g)	Total CTXs Mean ± SD (pg/g)
				Marakei Island				
Cephalopholis argus	001	M1	39	940	23.3	9.80	<LOQ	33.2
(Blue-spotted grouper)	002	M1	33	540	57.4	56.4	<LOQ	113
	003	M1	30	420	33.6	24.0	13.4	71.0
	004	M1	27	360	24.4	24.6	26.2	75.2
	005	M1	30	400	41.4	40.1	<LOQ	81.5
	006	M1	39	960	56.6	132	32.0	220
	007	M1	35	740	27.2	39.6	28.4	95.2
	008	M1	37	900	29.1	<LOQ	<LOQ	29.1
	009	M1	37	1260	26.2	48.2	<LOQ	74.4
	010	M1	30	440	25.7	55.5	<LOQ	81.9
	011	M1	33	700	35.7	<LOQ	<LOQ	35.7
	012	M4	27	360	<LOQ	<LOQ	<LOQ	<LOQ
	013	M1	40	1580	99.3	31.8	32.2	163
	014	M1	30	560	160	22.0	<LOQ	182
	015	M1	33	580	54.0	<LOQ	<LOQ	54.0
	016	M2	38	939	26.0	11.7	30.7	68.4
	017	M4	38	960	<LOQ	<LOQ	<LOQ	<LOQ
Cephalopholis aurantia	018	M4	35	647	10.1	12.7	7.10	30.6
(Golden hind)	019	M3	34	660	16.0	12.0	8.60	37.5
	020	M3	32	840	69.3	26.4	12.5	102
	021	M4	29	356	11.5	8.40	7.80	26.4
Cephalopholis urodeta	022	M1	18	120	20.7	8.70	<LOQ	29.4
(Darkfin hind)	023	M3	15	140	6.70	<LOQ	<LOQ	6.70
Epinephelus areolatus (Areolate grouper)	024	M4	28	284	<LOQ	<LOQ	<LOQ	<LOQ
Epinephelus coeruleopunctatus (White-spotted grouper)	025	M1	22	608	474	192	16.2	683
Epinephelus corallicola	026	M4	46	613	23.1	14.5	11.0	48.5
(Coral grouper)	027	M4	45	637	<LOQ	<LOQ	<LOQ	<LOQ
Epinephelus fuscoguttatus (Brown-marbled grouper)	028	M1	53	2520	298	94.9	48.0	441
Epinephelus hexagonatus (Starspotted grouper)	029	M2	30	348	<LOQ	<LOQ	<LOQ	<LOQ
Epinephelus macrospilos	030	M3	36	940	17.9	56.1	15.6	89.5
(Snubnose grouper)	031	M3	25	560	11.9	25.0	7.10	44.0

Epinephelus maculatus	032	M3	35	880	31.9	16.8	<LOQ	48.7
(Highfin grouper)								
Epinephelus merra	033	M1	17	78	<LOQ	<LOQ	<LOQ	<LOQ
(Honeycomb grouper)	034	M3	23	208	<LOQ	<LOQ	<LOQ	<LOQ
Epinephelus polyphekadion	035	M2	53	2621	32.8	22.9	9.90	65.8
(Camouflage grouper)	036	M1	51	2760	17.2	28.0	14.4	59.6
	037	M1	54	2800	7.90	7.90	<LOQ	15.8
Epinephelus tauvina	038	M1	51	2760	<LOQ	<LOQ	<LOQ	<LOQ
(Greasy grouper)	039	M1	54	2800	<LOQ	<LOQ	<LOQ	<LOQ
Lutjanus bohar	040	M2	46	1600	<LOQ	<LOQ	<LOQ	<LOQ
(Two-spot red snapper)	041	M2	57	4000	62.1	27.8	27.3	117
	042	M2	63	4400	24.8	10.1	15.6	50.5
	043	M2	48	1700	21.8	14.6	<LOQ	36.4
	044	M3	27	720	10.3	<LOQ	<LOQ	10.3
	045	M3	18	3600	355	90.0	75.0	520
Lutjanus fulvus	046	M1	19	230	<LOQ	<LOQ	<LOQ	<LOQ
(Blacktail snapper)	047	M3	20	300	<LOQ	<LOQ	<LOQ	<LOQ
				<u>Kiritimati Island</u>				
Cephalopholis argus	048	C1	24.5	1400	76.7	154	11.5	242
(Blue-spotted grouper)	049	C2	41.5	1800	9.20	3.80	<LOQ	12.9
	050	C2	34	1500	9.90	14.9	4.10	28.8
	051	C1	27.5	500	4.10	7.90	4.80	16.8
	052	C2	37	1300	<LOQ	<LOQ	<LOQ	<LOQ
	053	C1	37.5	1100	<LOQ	<LOQ	<LOQ	<LOQ
Cephalopholis miniate	054	C1	36.6	2000	38.7	156	87.1	282
(Coral hind)								
Lutjanus bohar	055	C2	53	3400	<LOQ	<LOQ	<LOQ	<LOQ
(Two-spot red snapper)								
Lutjanus fulvus	056	C2	22.5	600	<LOQ	<LOQ	<LOQ	<LOQ
(Blacktail snapper)	057	C2	22.2	202	5.90	<LOQ	<LOQ	5.90
	058	C1	18.7	103	<LOQ	<LOQ	<LOQ	<LOQ
	059	C1	26.5	1300	23.5	16.9	17.8	58.3
Lutjanus gibbus	060	C2	27.8	700	30.7	20.4	11.4	62.5
(Humpback red snapper)								

Table S2. Concentrations of CTXs of grouper and snapper from Marakei Island and Kiritimati Island determined by LC-MS/MS.

Species	number of individuals (n)	Sample Site	P-CTX-1		P-CTX-2		P-CTX-3		Total CTXs	
			Occur-rence	Mean Toxicity ± SD (pg/g)	Occur-rence	Mean Toxicity ± SD (pg/g)	Occur-rence	Mean Toxicity ± SD (pg/g)	Occur-rence	Mean Toxicity ± SD (pg/g)
<u>Marakei Island</u>										
Cephalopholis argus	17	M1, M2, M4	88.2%	42.4±38.2	70.6%	29.2±33.2	35.3%	9.60±14.0	88.2%	81.1±60.8
Cephalopholis aurantia	4	M3, M4	100%	26.7±28.5	100%	14.9±7.90	100%	9.00±2.40	100%	50.6±38.5
Cephalopholis urodeta	2	M1, M3	100%	13.7±9.90	50%	4.30±6.10	0%	ND	100%	18.1±16.0
Epinephelus areolatus	1	M4	0%	ND	0%	ND	0%	ND	0%	ND
Epinephelus coeruleopunctatus	1	M1	100%	474	100%	192	100%	16	100%	683
Epinephelus corallicola	2	M4	50%	11.6±16.3	50%	7.20±10.2	50%	5.50±7.70	50%	24.3±34.3
Epinephelus fuscoguttatus	1	M1	100%	298	100%	94.9	100%	48.0	100%	441
Epinephelus hexagonatus	1	M2	0%	ND	0%	ND	0%	ND	0%	ND
Epinephelus macrospilos	2	M3	100%	14.9±4.30	100%	40.5±22.0	100%	11.3±6.00	100%	66.7±32.3
Epinephelus maculatus	1	M3	100%	31.9	100%	16.8	0%	ND	100%	48.7
Epinephelus merra	2	M1, M3	0%	ND	0%	ND	0%	ND	0%	ND
Epinehelus polyphekadion	3	M1, M2	100%	19.3±12.6	100%	19.6±10.4	66.7%	8.10±7.40	100%	47.0±27.2
Epinephelus tauvina	2	M2	0%	ND	0%	ND	0	ND	0	ND
Lutjanus bohar	6	M2, M3	75%	79.1±137	66.7%	23.8±34.1	50%	19.6±29.3	75%	122±199
Lutjanus fulvus	2	M1, M3	0%	ND	0%	ND	0%	ND	0%	ND
Total			74.5%		63.8%		42.6%		74.5%	
<u>Kiritimati Island</u>										
Cephalopholis argus	6	C1, C2	66.7%	16.6±29.7	58.3%	30.2±61.2	50%	3.40±4.50	66.7%	50.2±95.0
Cephalopholis miniata	1	C1	100%	38.7	100%	156	100%	87.1	100%	282
Lutjanus bohar	1	C2	0%	ND	0%	ND	0%	ND	0%	ND
Lutjanus fulvus	4	C1, C2	50%	7.30±11.1	25%	4.20±8.50	25%	4.50±8.90	50%	16.0±28.3
Lutjanus gibbus	1	C2	100%	30.6	100%	20.4	100%	11.4	100%	62.5
Total			61.5%		53.9%		46.2%		61.5%	

Table S3. List of maximum length, trophic level, and food items of fish from FishBase (https://www.fishbase.se/search.php).

Species	Maximum length	Food items
Cephalopholis argus	60	Finfish, bony fish, nekton, polychaetes, worms, crustaceans and shrimps/prawns
Cephalopholis aurantia	60	Zoobenthos, nekton, finfish, bony fish, benth. crust.
Cephalopholis miniata	50	Zoobenthos, nekton, finfish, bony fish, shrimps/prawns and benth. crust.
Cephalopholis urodeta	28	Zoobenthos, nekton, finfish, bony fish, benth. crust., crabs and shrimps/prawns
Epinephelus areolatus	47	Zoobenthos, nekton, finfish, bony fish, benth. crust. and crabs
Epinephelus coeruleopunctatus	76	Nekton, finfish, zoobenthos, benth. crust. and bony fish
Epinephelus corallicola	49	NA
Epinephelus fuscoguttatus	120	Nekton, finfish, bony fish, zoobenthos, benth. crust., crabs, shrimps/prawns, and crustaceans
Epinephelus hexagonatus	27.5	Nekton, finfish, bony fish, zoobenthos, benth. crust., polychaetes, and worms
Epinephelus macrospilos	51	Zoobenthos, worms, polychaetes, benth. crust., cabs, nekton, finfish bony fish, mollusks, octopi, cephalopods, and squids/cuttlefish
Epinephelus maculatus	60.5	Zoobenthos, benth. crust., crustaceans, nekton, cephalopods, squids/cuttlefish, octopuses, nekton, finfish, and bony fish
Epinephelus merra	32	Nekton, finfish, zoobenthos, benth. crust., and bony fish
Epinephelus polyphekadion	90	Nekton, cephalopods, squids/cuttlefish, finfish, zoobenthos, benth.crust., crabs, lobsters, mollusks, gastropods, crustaceans, and bony fish
Epinephelus tauvina	100	Zooplankton, fish eggs/larvae, Chanos chanos , nekton, finfish, bony fish, zoobenthos, benth. crust. and shrimps/prawns
Lutjanus bohar	90	Zoobenthos, benth. crust., amphipods, shrimps/prawns, stomatopods, mollusks, gastropods, sponges/tunicates, ascidians, crabs, nekton and finfish
Lutjanus fulvus	40	Nekton, finfish, bony fish, cephalopods, squids/cuttlefish, crabs, benth.crust., zoobenthos, sponges/tunicates, echinoderms, worms, shrimps/prawns, sea cucumbers, stomatopods, amphipods, ascidians and non-annelids
Lutjanus gibbus	50	Zoobenthos, nekton, finish, bony fish, benth. crust., mollusks, worms, echinoderms, lobsters, crabs, shrimps/prawns, sea cucumbers, stomatopods, sea stars/brittle stars, polychaetes, other benth. Invertebrates, gastropods and non-annelids

NA: not available

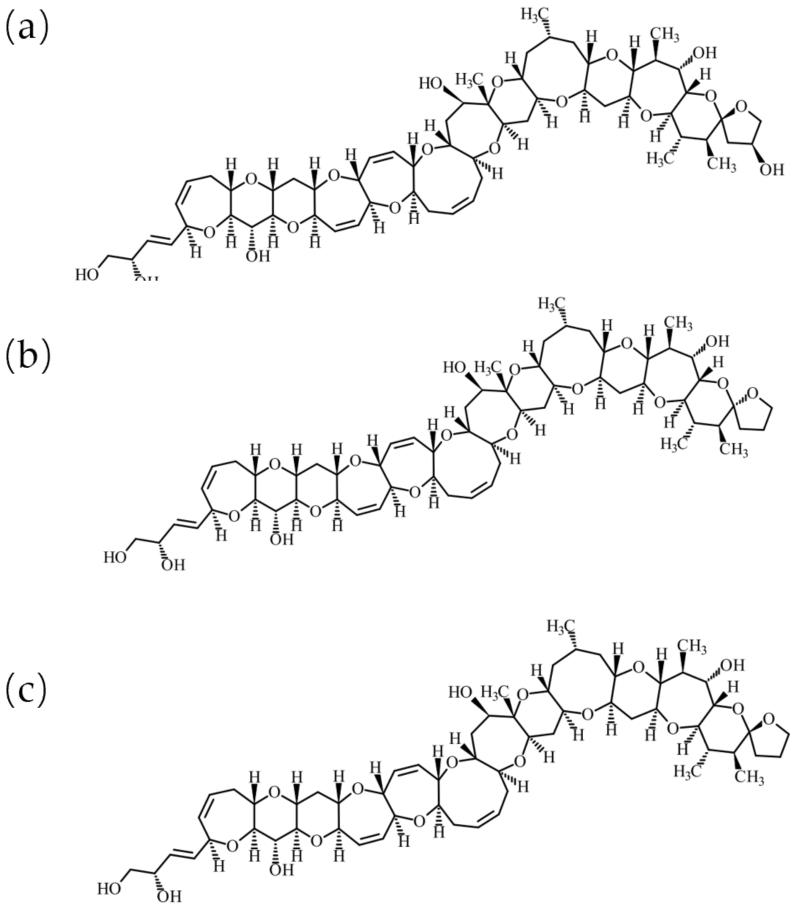

Figure S1. Chemical structures of (a) P-CTX-1, (b) P-CTX-2, and (c) P-CTX-3.

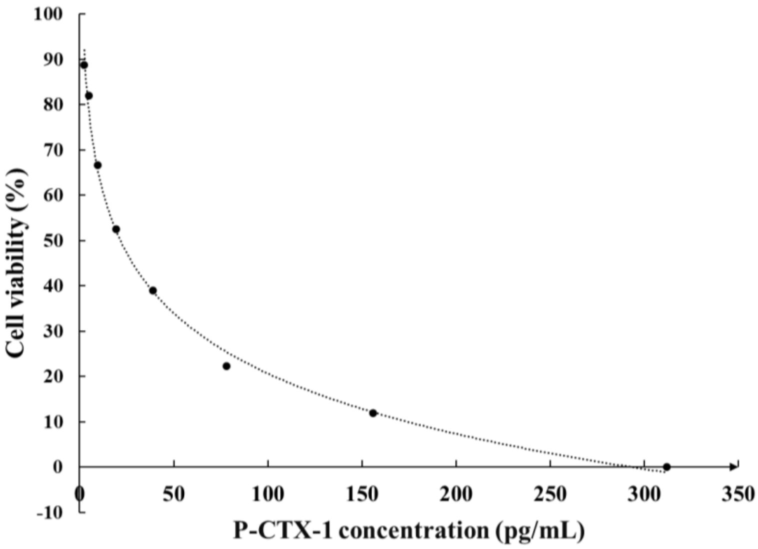

Figure S2. P-CTX-1 standard curve for N2a

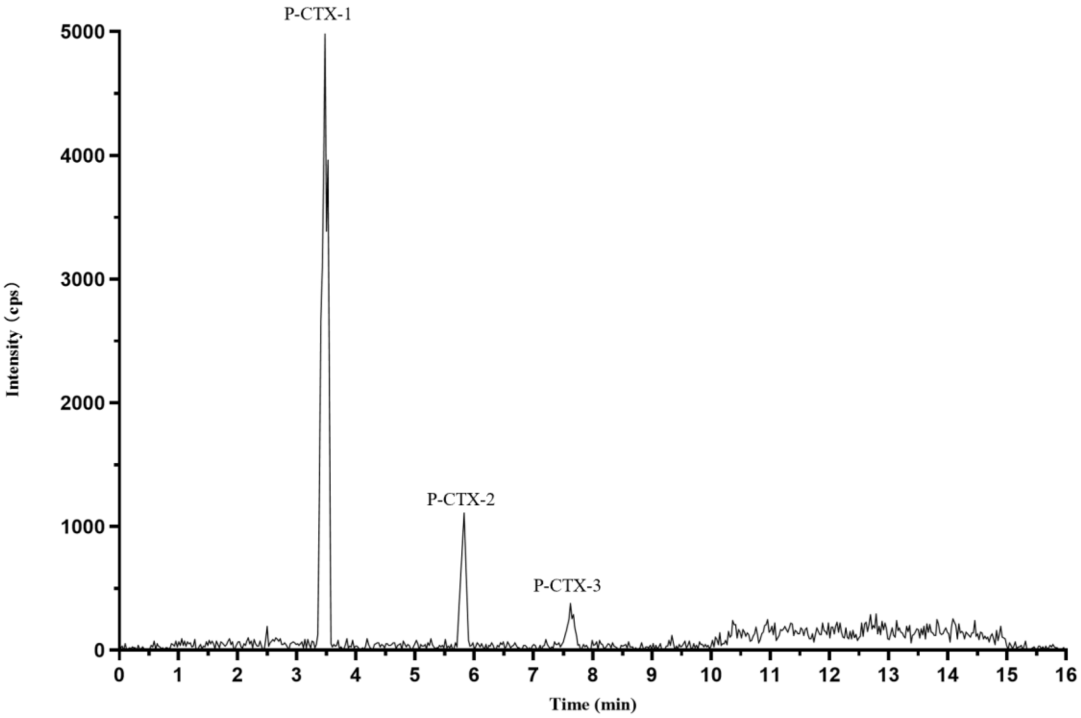

Figure S3. Representative chromatograms of contaminated sample after dilution (*Epinephelus coeruleopunctatus*).

Table S4. LC-MS/MS parameters and retention times for P-CTX-1, -2 and -3

Compound	Precursor ion (m/z)	Precursor ion (m/z)	Retention time	Delustering potential	Collision energy	Collision cell exit potential	Curtain gas	Ion Spray voltage	Temperature	Ion source gas 1	Ion source gas 2	Entrance potential
P-CTX-1	1128.4	1075.5*	3.48	51	29	24	10	5500	400	30	40	10
	1128.4	1093.5^	3.48	51	27	28	10	5500	400	30	40	10
	1128.4	1057.5^	3.48	51	31	16	10	5500	400	30	40	10
P-CTX-2	1112.7	1077.8*	5.83	51	25	20	10	5500	400	30	40	10
	1112.7	1059.8^	5.83	105	30	20	10	5500	400	30	40	10
	1112.7	1041.8^	5.83	105	30	20	10	5500	400	30	40	10
P-CTX-3	1112.7	1077.8*	7.62	51	25	20	10	5500	400	30	40	10
	1112.7	1059.8^	7.62	105	30	20	10	5500	400	30	40	10
	1112.7	1041.8^	7.62	105	30	20	10	5500	400	30	40	10

*= quantification ion; ^= confirmation ion
